# The effect of an e-intervention on intellectual disability stigma among Nigerian and Kenyan internet users: a comparative randomised controlled trial

**DOI:** 10.3389/fpsyt.2024.1331107

**Published:** 2024-03-05

**Authors:** Deborah Odukoya, Winfred Chege, Katrina Scior

**Affiliations:** Clinical Educational and Health Psychology, University College London, London, United Kingdom

**Keywords:** intellectual disability, stigma, attitudes, intervention, Africa, Kenya, Nigeria

## Abstract

**Introduction:**

The negative consequences of stigma for the wellbeing of people with disabilities have raised public and global health concerns. This study assessed the impact of an e-intervention to reduce intellectual disability (ID) stigma among Nigerian and Kenyan internet-users.

**Method:**

Participants aged 18+ and citizens of Nigeria and Kenya were recruited through online advertising. Qualtrics, a web survey platform, randomly assigned (1:1) participants to watch either a short experimental or control film, while masked to their assignment. The experimental film featured education about ID and indirect contact. The control film was on an unrelated topic. Their attitudes were measured on three dimensions (affect, cognitions and behaviour) at three time points (baseline, post intervention and one-month follow-up). Between October 2016 and April 2017, 933 participants were randomised, 469 to the experimental condition and 464 to the control condition. Of these, 827 (89%) provided pre-and post-intervention data but only 287 (31%) were retained at follow-up.

**Results:**

An intent-to-treat analysis revealed that participants in the experimental but not the control condition showed a positive shift in their attitudes towards people with ID over time. Their willingness to interact with people with ID increased post-intervention.

**Discussion:**

A brief intervention that integrates education and indirect contact can make an effective contribution to efforts to reduce stigma faced by people with ID in Africa. Trial registered with the ISRCTN trial registry (number ISRCTN92574712).

## 1 Introduction

People with intellectual disabilities (ID) are heavily stigmatised and marginalised globally (1–4). They are more likely to have their fundamental rights and freedoms denied. They are also more likely to experience higher levels of health, social and financial inequalities than their non-disabled counterparts (1, 3). Africa has been identified as one of the world regions where pejorative terminology, stigmatising beliefs and discriminatory practices towards people with ID continue to exist (3, 4). Misconceptions around the causes and abilities of people with ID have been identified as key drivers of stigma in African regions (1, 5, 6). Embedded cultural beliefs that disability is caused by spiritual forces or misdeeds from others, and a perception of people with ID as a burden to family/community resources have been identified in multiple studies (1, 5, 6). Such beliefs have resulted in people with ID becoming victims of ostracism, harmful traditional practices, violence and in some cases, death (5, 6). Accordingly, public health concerns regarding ID stigma have been voiced, resulting in a call for more global initiatives to challenge ID stigma particularly in low-income and middle-income countries (2, 5).

Documented efforts to reduce ID stigma in African countries have occurred at multiple levels, such as parent support and training groups, education campaigns, community-based rehabilitation programmes and mass media initiatives, with evidence of positive results in changing cultural beliefs and tackling discrimination (6–8). A community-based programme in Kenya promoted social inclusion and access to education for children with developmental disabilities by demonstrating their ability to learn through an 8-week motor skills programme (7). Another initiative in Egypt demonstrated changes in teachers’ attitudes by increasing knowledge and challenging misconceptions about ID as well as creating opportunities to work with people with ID in a sheltered workshop (8). While these initiatives appear promising, conceptual and methodological concerns regarding the case for their support have been noted (9). Furthermore, many persons with ID are yet to benefit from anti-stigma efforts due to huge disparities between world regions where high levels of stigma are prevalent and those where efforts to reduce ID stigma are mostly undertaken (2, 4). Accordingly, there is a need to do more to tackle societal barriers that impede the quality of life of people with ID in Africa and elsewhere (2, 4).

Contact and education have been identified as key routes to challenging stigma in other fields (10). In the case of ID, educational approaches challenge misconceptions and stereotypic myths about ID, and their benefits include their potential low cost and broad reach. However, for brief interventions, the effects of educational strategies vary in magnitude and duration and need to be combined with contact approaches to achieve longer-term change (10, 11). Contact approaches stem from Allport’s contact hypothesis (12) and propose that interactions between in-group members (i.e., those doing the stigmatising) and out-group members (i.e., those being stigmatised) can reduce prejudice, when certain conditions are met. These conditions, usually as part of direct contact, include one-to-one interactions with an out-group member of equal status, with intergroup cooperation, a pursuit of a common goal and support from authorities (12). A recent study in Kenya successfully used a contact-based approach to promote awareness of the capabilities of people with disabilities (ID included) and challenge dehumanisation. This was done by positioning people with disabilities as agents of change and supporting them in voicing their own narratives as ‘experts by experience’ (13).

However, securing direct contact on a large scale can prove difficult and costly and can limit control over quality. Furthermore, in light of growing evidence it is now known that while Allport’s key conditions enhance the positive effects of contact approaches, they are not required to produce positive outcomes (12). Research has shown that the level of positive outcomes achieved in any contact situation is based on the extent to which the exposure reduces threat and anxiety about future intergroup contact, while also inducing positive affect such as empathy (12, 14).

Indirect contact with people with ID, via film footage as a standalone intervention or as a component of a multi-faceted anti-stigma programme, is scalable and a viable way to control for some of the potential drawbacks of direct contact (14). Indirect contact through film seeks to achieve change in three major ways: 1) by creating an experiential learning situation, 2) eliciting empathy, and 3) encouraging inferential processes in the viewer (14). Several studies have tested the impact of brief digital interventions and found that indirect contact is an effective way to change attitudes towards people with ID among the general public (14–16).

However, it has been reported that intermittent connectivity problems in Africa may serve as a barrier in the deployment and uptake of digital initiatives that deliver film-based contact (17). Despite these concerns, there is growing evidence that Nigeria and Kenya are increasingly recognising the need to establish strong information and communication technologies (ICTs) for health initiatives. In the Kenyan National e-Health Strategy, providing equitable and affordable healthcare at the highest achievable standard to Kenyan citizens was listed as a main goal. E-learning was identified as a key strategic area of implementation (18). Also, according to the United Nations Foundation, the Nigerian government has formally recognised the importance of ICTs to improve access to health services and interventions (16). As such, looking at the effectiveness and appropriateness of digital integrated approaches to reduce stigma warrants more attention in African countries. Digital approaches may not only provide a viable medium to carry out anti-stigma initiatives but also align with the ICT agenda put forth by the Nigerian and Kennya governments respectively. The present study aimed to test the effectiveness of an e-intervention integrating education and indirect film-based contact in raising awareness about ID and reducing public stigma in Nigeria and Kenya.

## 2 Methods

### 2.1 Study setting, design, and participants

In this randomised controlled trial (RCT), a film-based intervention was delivered to Nigerian and Kenyan internet users and its efficacy tested using repeated measures. The intervention was produced in collaboration with non-governmental organisations in Nigeria and Kenya. Qualtrics, a web survey platform, was used to randomly assign participants to the experimental or control condition and to collect data. Data were collected at three time points: baseline, immediately post film, and one-month follow-up to allow estimation of the size of any effects and assessment of any lasting positive effects. The trial was registered with the ISRCTN trial registry (ISRCTN92574712).Eligible participants were at least 18 years of age, English speaking, and Nigerian/Kenyan internet users. Participants who did not meet the above criteria were excluded. All participants were recruited through social media advertising (Instagram, Facebook and email promotions), containing brief details of the study, and a link to the data collection site. They were informed about their right to withdraw from the study at any time, and that starting the survey would be taken as informed consent. No adverse effects were reported by participants.

### 2.2 Randomisation and masking

Participants were randomly assigned on a 1:1 ratio to the experimental group or the control group using a block randomisation code embedded within Qualtrics. Enrolment, generation of sequences and assignment of participants were all pre-programmed. Participants were informed that the aim of the study was to gather public opinions regarding personal difficulties some people face. They were not aware that the study’s primary objective was to measure potential attitude change. Both groups were presented with the same information and outcome measures. The only difference between groups was the content of the video shown after participants had completed the baseline survey. Before the baseline measures, participants were provided with a brief description of ID to ensure that they had an adequate understanding of the condition as basis for completing measures on their attitudes to ID. The description was as follows:

For the purpose of this study, intellectual disability is a term used when a person has certain delays in their cognitive development. These delays must be present before the person reaches adulthood and can lead to difficulties understanding, learning and remembering new things. It may also affect the person’s communication, social and self-care skills. A person with an intellectual disability may therefore develop and learn more slowly or differently than others. In the past, the term ‘mental retardation’ was used to describe intellectual disability. Some specific syndromes and conditions like Fragile X and autism may in some cases be associated with having an intellectual disability. Intellectual disabilities are different from specific learning difficulties such as dyslexia, which are NOT the focus of this study.

To reduce ascertainment bias and ensure blinding, the control group watched a documentary film of a similar length and structure to the experimental group. Dropout rates between groups after watching the film-intervention were compared to assess the success of masking. Investigators were not blinded to the intervention.

### 2.3 Procedure

Once participants had completed the baseline measures, they were randomised to one of two film conditions in each study. Participants in the experimental group watched a 6-minute film providing information about ID and its causes and consequences, countering stigmatising beliefs known to be common in Africa, and indirect contact. Stigmatising beliefs targeted in the film were based on a global review conducted on ID stigma (4). Also, all stages in the development of the film were reviewed by experts, researchers and representatives of organizations/advocacy networks in the ID field in Nigeria and Kenya. Some of them also held dual roles as parents of people with ID. The length of the film was determined by reviewing what similar studies had found to be effective (14, 19). The educational segment of the film was structured based on Leventhal’s Common Sense Model of how illness is conceptualised within the general population (20). This model proposes that five main components make up our representation of illnesses and influence our perceptions, attitudes and actions towards different illnesses. These include identity, cause, timeline, consequence and curability/controllability. As such, the selection of factual knowledge delivered was guided by identity (What is ID and what isn’t)?, causes (What causes ID and what doesn’t), timeline/curability (Is there a ‘cure’)?, and consequence (How might having an ID impact on someone’s abilities)? (20). This model has been used successfully in past anti-stigma initiatives as a framework for how factual information regarding ID is shared (19). This section of the film was delivered by local experts, to ensure its credibility. Experts were chosen based on their level of experience and involvement with families and individuals with ID locally. Two experts (a community paediatrician and the president of the Down Syndrome Foundation Nigeria) ran ID learning centres in their local community that focused on education and social care; another was a religious leader and one a psychiatrist, all with frequent contact with people with ID.

The indirect contact section featured people with moderate ID who varied in life roles and the challenges they faced, talking about their experiences, demonstrating their capabilities and talking about their hopes and aspirations. It also highlighted the magnitude of stigma they face in their respective countries. Separate but similar films were produced for the Nigerian and Kenyan studies to ensure credibility of both the experts and people with ID. The authors met and heard first-hand experiences from local people with ID and collaborated with them on how to create a film that would help change public misconceptions.

Written informed consent was obtained from all people featured and their parents for the recording and sharing of the film. An easy read version of the consent form, which included shorter sentences and images, was available for people with ID. The films are publicly viewable on YouTube: https://www.youtube.com/watch?v=2MpipkGk9Zs (Nigeria) and https://www.youtube.com/watch?v=ZSi_DJxGPrs&t=99s (Kenya).

The control group watched a film that was unrelated to ID, which focused on the challenges children in Kenya/Nigeria face in receiving an education. It had a similar length and format to the experimental video. It showed an expert talking about the education crisis in the respective country and demonstrated its impact on children. This film was chosen to control for the following variables that might influence observed change: reactivity to the outcome measures, study participation, length of film, and the social and demographic characteristics of people featured in the film. A feature on Qualtrics, known as force response, was embedded after both films to ensure that all participants watched the films before progressing to the next part of the study. Following the film, participants completed post-intervention measures and, if consenting, were contacted by email asking them to complete the follow-up survey a month later. Given the novelty of conducting an online longitudinal study within an African population, retention strategies were used to try to minimise participants’ attrition rates. This included the use of non-monetary incentives (i.e., gift vouchers) and reminder emails with patients’ consents. Local experts in the field were consulted regarding what incentives would be most attractive in the local context. Steps were also taken to prevent multiple submissions by embedding an “end of survey” function in Qualtrics. This function ensured that any attempts to retake the questionnaire on a browser or device that had previously been used was flagged and stopped.

### 2.4 Measures

The Attitudes towards Intellectual Disabilities (ATTID) scale, which draws on a multi-dimensional understanding of attitudes was used as the primary outcome measure in both countries (21, 22). The ATTID assesses the cognitive, affective, and behavioural components of attitudes across five-factors: two factors (*Discomfort* and *Sensitivity/Tenderness*) in the affective dimension; two factors (*Knowledge of Causes* and *Knowledge of Capacity and Rights*) in the cognitive dimension*;* and one factor (*Interaction*) in the behavioural dimension. The affective and behavioural dimensions of the scale are measured using two vignettes that present two men with ID, one with a higher and the other with a lower level of functioning. This study used the ATTID short form which consists of 36 items, using a 5-point Likert scale (1 = agree completely to 5 = disagree completely; plus an option of 9 to indicate “I don’t know”/”not applicable”). Its psychometric properties were examined for both the Nigerian and Kenyan data sets, yielding a six-factor structure for both, with three factors loading on the cognitive dimension instead of two as in the original Canadian sample (*Knowledge of Causes*, *Knowledge of Capacity*, and *Knowledge of Rights*) but an otherwise identical factor structure. The short version showed acceptable to good internal reliability with Cronbach’s alphas ranging from 0·68 to 0·88 for the six factors.

The causal beliefs listed in the ATTID were supplemented with three items from the supernatural causes subscale of the Intellectual Disabilities Literacy Scale (IDLS) to tap into superstitious causal attributions common in African countries and implicated in ID stigma (23). These items addressed ID potentially being seen as due to a test from God/Allah, possession by spirits, and punishment for past wrongdoings. This IDLS subscale has previously been tested in a range of cultural contexts, showing high internal (α= 0·76) and acceptable test-retest reliability (>0·7) (23).

Socio-demographic data (age, gender, ethnicity, religious affiliation, educational attainment, and prior contact with someone with ID) were also recorded at the end of the post-intervention survey.

### 2.5 Statistical analysis

An *a priori* power analysis completed using G*Power 3·1·8 (24), indicated a sample of 398 participants for each of the two studies (199 per group) to ensure an 80% chance (alpha set at 0·05) of detecting a ‘small’ effect of *d*= 0·25 as observed in a similar previous study (14) when comparing two independent means. Separate intention-to-treat analyses including all randomised participants were computed for the Nigerian and Kenyan samples using SPSS version 22. Assumptions of normality, linearity, multicollinearity and homoscedasticity were checked to ensure no violation. To assess the pattern of missing data due to participant drop-out, Little’s MCAR test was carried out, which showed that data were missing at random: χ^2^ (273, N=571)= 291·80, *p*= 0·207 (Nigerian study); *x²* (39, N=457) = 40·45, *p* =·406 (Kenyan study). As such, intervention effects were analysed using a linear mixed model. This model is a superior way to handling missing data in RCTs, outperforming other traditional methods; it uses all data presented at each time point and does not rely on complete cases to run analyses (25). For all analyses, p values of <0·01 were considered significant to manage the risk of type 1 error. Effect sizes were calculated following Morris’ guidelines for repeated measures control group designs (26).

### 2.6 Ethical considerations

The authors assert that all procedures contributing to this work comply with the ethical standards of the relevant national and institutional committees on human experimentation. All procedures involving human patients were approved by the authors’ institutional research ethics committee (ID: 8807/001). Written informed consent was provided by all persons who participated in the study.

## 3 Results

Participants were recruited between October 26, 2016 and April 28, 2017. In the Nigerian study, a total of 917 participants visited the survey site. Three did not meet the study’s inclusion criteria and were excluded. Of the remaining 914 participants, 215 (23·5%) dropped out after reading the information sheet and before beginning the study. Of the 699 that started, 571 (81·6%) completed the survey assessing baseline attitudes and were subsequently randomised (291 to the experimental group and 280 to the control group). A further 64 (9·2%) participants from both groups dropped out during the post-intervention survey. Another 311 (44·5%) dropped out between post-intervention and follow-up; of these participants, 51(7·3%) declined being contacted for the follow-up survey.

**Figure 1:**
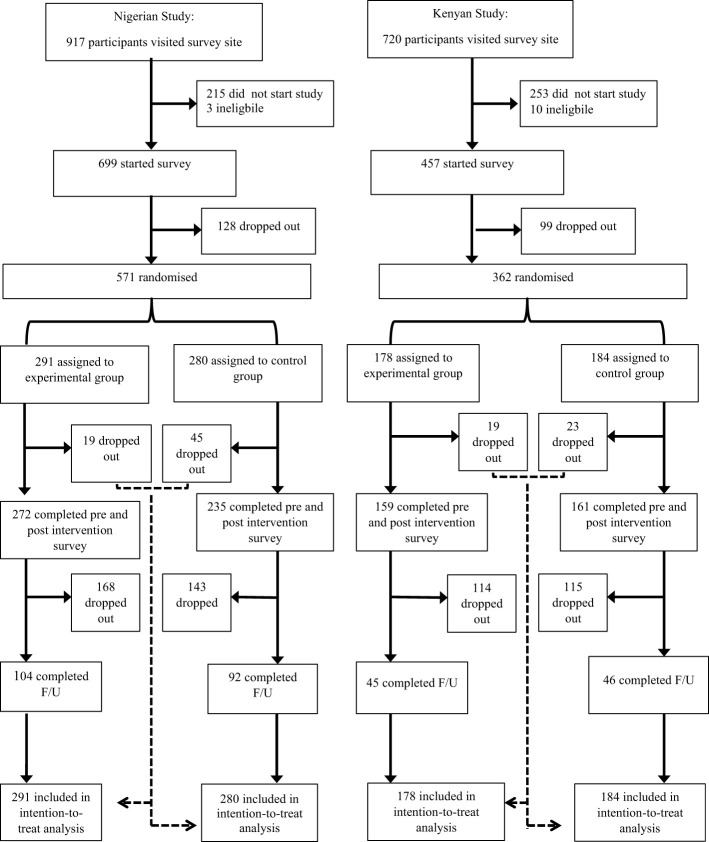
Trial profile. All dropouts occurred during transition points in the study. This included starting a vignette in the ATTID at time point 1, 2 and 3, during the film and the time period between post-intervention and follow-up.

In the Kenyan study, a total of 720 participants visited the survey site. Ten did not meet the inclusion criteria and 253 (35·6%) dropped out before beginning the study. Of the 457 that started the study, 362 (79·2%) completed the survey assessing baseline attitudes and were subsequently randomised (178 to the experimental group and 184 to the control group). During the post-intervention survey, 42 (9·2%) dropped out. A further 229 (50·1%) dropped-out between post-intervention and follow-up, including three participants who declined being contacted for the follow-up survey. The intention-to-treat analysis contained all 571 Nigerian and 418 Kenyan randomised participants (**Figure 1**).

In both studies, participants who completed the study were predominantly female, aged 25 to 34, and Christian, with a university/postgraduate degree (**Table 1**).

**Table 1:** Participant Characteristics.

	Nigerian Study		Kenyan Study	
	Intervention group (n=272) n (%)	Control group (n=229) n (%)	Intervention group (n=158) n (%)	Control group (n=161) n (%)
Sex*				
Male	62 (22%)	55 (24%)	48 (31%)	47 (29%)
Female	210 (77%)	173 (76%)	110 (70%)	114 (71%)
Missing	0	1 (0%)	0	0
Age (years) *				
18-24	49 (18%)	41 (18%)	14 (9%)	20 (12%)
25-34	153 (56%)	130 (57%)	109 (69%)	103 (64%)
35-44	52 (19%)	39 (17%)	15 (9%)	18 (11%)
45+	18 (7%)	18 (8%)	20 (13%)	20 (13%)
Education*				
Primary/Secondary	10 (4%)	12 (5%)	3 (2%)	10 (6%)
University	153 (56%)	122 (53%)	83 (53%)	90 (56%)
Post-graduate	109 (40%)	95 (42%)	61 (39%)	53 (32%)
Vocational qualification (Kenya Only)			11 (7%)	8 (5%)
Religion*				
Christian	270 (99%)	217 (95%)	143 (90%)	128 (80%)
Muslim	1 (0%)	9 (4%)	2 (1%)	8 (5%)
Hindu/Buddhist	0	0	2 (1%)	3 (2%)
Traditional religion	0	1 (0%)	1 (1%)	3 (2%)
Non-religious	1 (0%)	2(1%)	10 (6%)	19 (12%)
Prior Contact*				
Yes	191 (71%)	151 (66%)	128 (81%)	127 (79%)
No	80 (30%)	77 (34%)	30 (19%)	34 (21%)
Missing	1 (0%)	1 (0%)	0	0
Nature of Contact*				
Family member	44 (16%)	37 (16%)	48 (30%)	45 (28%)
Friend/Neighbour	57 (21%)	30 (13%)	36 (23%)	35 (22%)
Professional/Educational	35 (13%)	35 (15%)	17 (11%)	29 (18%)
Acquaintance/secondary relationships	42 (15%)	33 (14%)	21 (13%)	12 (7%)
Multiple Relationships	8 (3%)	6 (3%)	3 (2%)	2 (1%)
Stranger	3 (1%)	9 (4%)	3 (2%)	2 (1%)
Missing	3 (1%)	2 (1%)	0	2 (1%)
Not applicable	80 (29%)	77 (34%)	30 (19%)	34 (21%)
Frequency of contact*				
Weekly	50 (18%)	31 (14%)	19 (12%)	22 (14%)
Several times a month but < weekly	26 (10%)	16 (7%)	14 (9%)	16 (10%)
Occasionally during the year	58 (21%)	47 (21%)	56 (35%)	45 (28%)
Less than 1x a year	21 (8%)	22 (10%)	38 (24%)	43 (27%)
A one-off encounter	32 (12%)	33 (14%)	0	0
Missing	5 (2%)	3 (1%)	1 (1%)	1 (1%)
Not applicable	80 (29%)	77 (34%)	30 (19%)	34 (21%)

* Due to attrition, demographic information collected at the end of the survey does not include all participants included in the intention-to-treat analysis.

Descriptive data for both groups are presented in **Table 2**. A series of linear mixed models were computed to test for intervention effects across all six ATTID subscales and the IDLS supernatural causal beliefs subscale. In the Nigerian study, significant time x group interactions were found for the affective attitude dimensions *Discomfort* and *Sensitivity*; for the behavioural dimension *Interaction*; and for two of the three cognitive dimensions, *Knowledge of Rights* and Knowledge of *Capacity*, but not for *Knowledge of Causes*. Significant interactions were also found for *Supernatural Beliefs* (**Table 3**). In the Kenyan study, significant time x group interactions were observed for all ATTID subscales, except for *Knowledge of Rights.* No significant interactions were found for the *Supernatural Beliefs* subscale (**Table 3**). The significant interactions indicated that there were substantial differences in attitude scores between groups over time.

**Table 2:** Attitude subscale scores by time point and study: Means (standard deviations).

	Nigerian Study						Kenyan Study					
	Treatment group			Control group			Treatment group			Control group		
	Pre	T1	FU	Pre	T1	FU	Pre	T1	FU	Pre	T1	FU
ATTID Subscales												
Discomfort	2.58 (0.80)	2.16 (0.76)	2.40 (0.86)	2.54 (0.82)	2.49 (0.83)	2.39 (0.79)	2.39 (0.79)	1.95 (0.79)	2.08 (1.01)	2.39 (0.82)	2.29 (0.78)	2.14 (0.65)
Sensitivity	3.68 (0.75)	3.36 (0.85)	3.50 (0.77)	3.68 (0.73)	3.60 (0.83)	3.51 (0.84)	3.57 (0.74)	3.05 (0.90)	3.05 (1.02)	3.54 (0.81)	3.35 (0.85)	3.32 (0.77)
Interaction	2.56 (0.64)	2.21 (0.63)	2.39 (0.67)	2.50 (0.64)	2.46 (0.67)	2.48 (0.66)	2.62 (0.75)	1.88 (0.78)	2.33 (1.34)	2.48 (0.52)	2.40 (0.49)	2.51 (0.61)
Knowledge of Rights	1.91 (0.72)	1.83 (0.69)	1.93 (0.67)	1.85 (0.78)	2.03 (0.76)	2.10 (0.86)	1.75 (0.74)	1.69 (0.68)	1.95 (0.69)	1.81 (0.71)	1.91 (0.76)	1.91 (1.12)
Knowledge of Capacity	2.81 (0.75)	2.13 (0.68)	2.47 (0.79)	2.91 (0.79)	2.71 (0.78)	2.75 (0.66)	2.54 (0.48)	2.29 (0.50)	2.43 (0.77)	2.61 (0.79)	2.33 (0.84)	2.71 (0.79)
Knowledge of Causes	2.38 (0.63)	2.37 (0.71)	2.36 (0.72)	2.39 (0.65)	2.41 (0.74)	2.47 (0.71)	2.24 (0.59)	2.16 (0.76)	2.33 (0.82)	2.24 (0.68)	2.32 (0.79)	2.23 (0.62)
IDLS: Supernatural Causal Beliefs	2.22 (0.89)	1.78 (0.78)	1.85 (0.85)	2.14 (0.85)	1.97 (0.87)	1.95 (0.86)	1.82 (0.06)	1.45 (0.06)	1.54 (0.11)	1.82 (0.06)	1.78 (0.06)	1.93 (0.10)

**Table 3:** Results of linear mixed models testing for time x group interactions.

	Nigerian Study		Kenyan Study	
	F	p	F	p
ATTID Subscales				
Discomfort	*F* (2,750) = 21.23	<0.001**	*F*(2, 443) = 12.67	<0.001**
Sensitivity	*F* (2,750) = 11.13	<0.001**	*F(*2, 445) = 11.57	<0.001**
Interaction	(*F* (2,751) = 23.14	<0.001**	*F*(2, 488) = 13.95	<0.001**
Knowledge of Rights	*F* (2,791) = 10.74	<0.001**	*F*(2, 473) = 3.42	0.033
Knowledge of Capacity	*F* (2, 841) = 23.84	<0.001**	*F*(2,444) = 6.45	0.002*
Knowledge of Causes	*F* (2, 796) = 0.69	0.503	*F* (2, 466) = 4.97	0.007*
IDLS: Supernatural Causal Beliefs	*F* (2,784) = 11.13	0.001*	*F*(2, 463) = 2.49	0.084

*p <.01, **p <.001.

*Post hoc* analyses of least significant difference (LSD) comparisons were then carried out for subscales that showed significant interactions in order to determine the exact nature of attitude change. In the Nigerian experimental group, LSD comparisons showed a significant reduction in negative attitudes from pre-to post-intervention for the following factors: *Discomfort*, *Sensitivity*, *Interaction*, *Knowledge of Rights*, *Knowledge of Capacity*, and *Supernatural Beliefs*. These favourable changes were all maintained at follow-up when compared to baseline with the exception of *Sensitivity* and *Knowledge of Rights* (**Table 4**). The positive shifts from baseline to follow-up were medium to large for *Knowledge of Capacity* (*d* = -0·624), small for *Interaction* (*d* = -0·234), *Supernatural Beliefs* (*d* = -0·206) and *Discomfort* (*d* = -0·163).

**Table 4:** Results of *post hoc* analyses for subscales showing significant interactions (Nigerian Study).

	Intervention								Control								
	Pre - Post				Pre - FU				Pre - Post				Pre - FU				
	*b (CI)*	*t*	*df*	*p*	*b (CI)*	*t*	*df*	*p*	*b (CI)*	*t*	*df*	*p*	*b (CI)*	*t*	*df*	*p*	
ATTID Subscales																	
Discomfort	0.40 (0.32 - 0.48)	10.42	279	<0.001**	0.20 (0.08–0.33)	3.14	148	0.002**	0.05 (-0.02– 0.11)	1.39	243	0.166	0.05 (-0.07 – 0.17)	0.83	112	0.408
Sensitivity	0.31 (0.24 - 0.39)	8.49	279	<0.001**	0.19 (0.07- 0.32)	2.99	152	0.030	0.63 (0.00-0.13)	1.20	245	0.047	0.15 (0.02-0.27)	2.36	127	0.020
Interaction	0.34 (0.28 - 0.40)	11.52	279	<0.001**	0.22 (0.12-0.32)	4.45	145	<0.001**	0.05 (-0.01- 0.10)	1.69	245	0.093	0.03 (-0.08- 0.14)	0.60	132	0.550
Knowledge of Capacity	0.68 (0.59-0.78)	13.63	289	<0.001**	0.39 (0.25-0.53)	5.29	194	<0.001**	0.19 (0.09- 0.29)	3.63	265	<0.001**	0.12 (-0.03-0.27)	1.62	158	0.107
Knowledge of Rights	0.85 (0.02 - 0.15)	2.42	285	<0.016*	0.37 (-0.09- 0.16)	0.58	167	0.560	-0.16 (-0.23–0.84)	4.24	259	<0.001**	-0.20 (-0.34–0.58)	2.79	154	0.006*
IDLS: Supernatural Causal Beliefs	0.44 (0.36 - 0.53)	10.0	286	<0.001**	0.36 (0.22 - 0.50)	5.37	151	<0.001*	0.17 (0.08 -0.26)	3.98	258	<0.001**	0.08 (-0.06-0.23)	1.15	123	0.253

*p <.01, **p <.001.

In the Nigerian control group, LSD comparisons revealed no significant change in *Discomfort, Sensitivity*, and *Interaction* over time. However, there was a significant positive shift in attitudes observed from pre-to post-intervention for *Knowledge of Capacity*, and *Supernatural Beliefs* (**Table 4**). These changes were not maintained at follow-up. In addition, a significant negative shift in *Knowledge of Rights* was observed in the control group post-intervention and maintained at follow-up.

The observed pre-post reductions in negative attitudes were consistently larger in the Nigerian experimental group when compared to the control group (**Table 2**). An interaction analysis comparing the Nigerian experimental and control groups from pre-to post-intervention showed significant effects for *Discomfort*, *Sensitivity*, and *Interaction* (**Table 5**).

**Table 5:** Interaction analysis comparing attitudes by group: baseline to post-intervention.

	Nigeria Pre to Post x group				Kenya Pre to Post x group			
	*b (CI)*	*t*	*df*	*p*	*b (CI)*	*t*	*df*	*p*
ATTID Subscales								
Discomfort	0.31 (0.18-0.45)	3.53	515	<0.001**	0.30 (0.14-0.47)	3,53	515	<0.001**
Sensitivity	0.25 (0.12-0.38)	3.10	517	0.002*	0.27 (0.10-0.45)	3.10	517	0.002*
Interaction	0.23 (0.12-0.34)	5.57	612	<0.001**	0.46 (0.30-0.62)	5.57	612	<0.001**
Knowledge of Capacity	0.25 (0.12-0.38)	2.10	513	0.036	0.11 (0.01-0.23)	2.10	513	0.036
Knowledge of Rights	0.19 (0.06-0.31)	2.46	554	0.014	0.20 (0.05-0.36)	2.54	528	0.011
Knowledge of causes	0.03 (-0.08- 0.15)	0.65	515	0.515	0.14 (-0.01-0.29)	1.79	519	0.075
IDLS: Supernatural Causal Beliefs	0.18 (0.04-0.32)	2.53	833	<0.012	-0.25 (-0.44 - -0.06)	-2.63	512	0.009*

*p <.01, **p <.001.

In the Kenyan study, *post hoc* LSD comparisons in the experimental group showed significant reductions in negative attitudes from pre-to post-intervention for the following factors: *Discomfort*, *Sensitivity*, *Interaction*, and *Knowledge of Capacity*. *Knowledge of Rights* and *Knowledge of Causes* showed no significant change post-intervention. Looking specifically at the significant positive changes observed, these were maintained at follow-up when compared to baseline for *Discomfort*, *Sensitivity*, and *Interaction* but not for *Knowledge of Capacity* (**Table 6**). The positive shifts from baseline to follow-up were medium sized for *Interaction* (-0.489), small to medium for *Sensitivity* (-0.388), and negligible for *Discomfort* (-0.075).

**Table 6:** Results of *post hoc* analyses for subscales showing significant interactions (Kenyan Study).

	Intervention								Control								
	Pre - Post				Pre - FU				Pre - Post				Pre - FU				
	*b (CI)*	*t*	*df*	*p*	*b (CI)*	*t*	*df*	*p*	*b (CI)*	*t*	*df*	*p*	*b (CI)*	*t*	*df*	*p*	
ATTID Subscales																	
Discomfort	0.44 (0.33-0.56)	7.72	166	<0.001**	0.34 (0.12-0.56)	3.08	83	0.003*	0.07 (-0.01-0.16)	1.79	166	0.075	0.22 (0.05-0.39)	2.64	51	0.011
Sensitivity	0.52 (0.41-0.64)	8.75	166	<0.001**	0.55 (0.32-0.77)	4.81	111	<0.001**	0.17 (0.09-0.26)	4.23	166	<0.001**	0.14 (-0.02-0.30)	1.79	59	0.079
Interaction	0.71 (0.60-0.84)	12.11	170	<0.001**	-0.31 (0.12-0.51)	3.17	73	<0.002*	0.27 (0.15-0.40)	4.25	179	<0.001**	-0.12 (-0.33-0.10)	1.10	99	0.276
Knowledge of Capacity	0.24 (0.17-0.31)	7.05	165	<0.001**	0.10 (-0.02-0.22)	1.62	73	0.109	0.08 (0.03-0.14)	3.08	167	0.002*	-0.12 (-0.12-0.08)	0.38	65	0.706
Knowledge of Rights	0.04 (-0.06-0.15)	0.83	170	0.410	-0.19 (-0.38 - -0.01)	-2.09	67	0.040	-0.11 (-0.21-0.02)	2.35	177	0.020	-0.10 (-0.29-0.09)	1.06	86	0.292
Knowledge of Causes	0.87 (-0.01-0.19)	1.69	170	0.090	-0.99 (-0.27 - -0.09)	-0.09	91	0.322	-0.09 (-0.17-0.12)	2.29	176	0.023	-0.00 (-0.13-0.13)	0.01	62	0.990

*p <.01, **p <.001.

In the Kenyan control group, *Discomfort* and *Supernatural Beliefs* showed no significant pre-to post intervention change but a reduction in negative attitudes was observed for *Sensitivity*, *Interaction*, and *Knowledge of Capacity*. However, these changes were not maintained at follow-up. Similar to the Nigerian study, a significant baseline to post-intervention increase in negative attitudes was found in the Kenyan control group for *Knowledge of Rights*, but also *Knowledge of Causes*, although none of these changes were maintained at follow-up.

The baseline to post-intervention reductions in negative attitudes observed in the Kenyan study were consistently larger in the experimental group when compared to the control group (**Table 2**). Similar to the Nigerian study, an interaction analysis comparing the two groups showed significant effects for *Discomfort*, *Sensitivity*, and *Interaction* (**Table 5**).

## 4 Discussion

The present study set out to investigate the effectiveness of an e-intervention that integrated education and indirect contact to challenge public stigma associated with ID in Nigeria and Kenya. It distinguished and measured all three components of attitudes (cognition, affect and behavioural intention) in order to adequately assess attitude change. Our key findings were: (1) the experimental group in both Nigeria and Kenya on average showed a small to medium positive shift in participants’ affect and behavioural intentions, which were maintained at 1-month follow-up expect for the *Sensitivity* subscale in the Nigerian study; (2) both studies also showed a change in participants’ beliefs regarding capacity, however, this shift was only maintained in the Nigerian study at follow-up; (3) only the Nigerian study showed a shift in supernatural causal beliefs, which was maintained over time (4) the Nigerian study also showed a shift in participants’ knowledge of rights but this change was not maintained over time; (5) neither study showed changes in participants’ knowledge of causes; and (6) all observed changes were statistically superior in the intervention group in comparison to the control group.

The use of an online platform to disseminate anti-stigma interventions raises questions around two competing agendas: population penetration versus level of impact (11). Film-based (indirect) contact allows for dissemination through multiple media channels leading to larger audiences for anti-stigma interventions. On the other hand, direct contact yields better intervention effects due to its ability to promote more personalised, targeted efforts (11). As opposed to the medium to large effects often reported in response to direct contact, the magnitude of change observed in this study was mostly within the small to medium range which is consistent with other indirect contact studies (11, 14). However, the number of people reached through the present study was exponentially larger when compared to other non-government led anti-stigma efforts in Africa (4, 7, 8). To date, most interventions coming out of African regions are grassroot efforts that are mostly limited in duration, size and impact due to very limited resources (1, 2). This is not for a minute to diminish the value of local grassroots efforts but to highlight a parallel need for cost-effective anti-stigma initiatives that have the potential for population penetration. This is particularly important given that public awareness and acceptance play an important role in encouraging community participation for stigmatised individuals (1). Having said that, the use of an integrated approach of education and indirect contact may offer an avenue for larger impact within public stigma efforts. The Nigerian study showed a medium to large effect on *Knowledge of Capacity* which was maintained over time, a magnitude of change that is more commonly seen for standalone direct contact efforts (11). However, this effect was not replicated within the Kenyan study and this difference between both countries may offer insight to important mechanisms of change.

Some noticeable differences in attitude change post-intervention were observed between the two countries. While in the Nigerian study endorsement of stigmatising supernatural causal beliefs decreased in response to the intervention, the experimental film had no effect on *Supernatural Beliefs* in the Kenyan study. This may have been due to the Kenyan sample’s much greater endorsement of supernatural beliefs at baseline compared to the Nigerian sample. Also, a slightly higher number of participants were affiliated to a religion in the Nigerian study compared to the Kenyan study which had more participants that identified as non-religious. While the difference was small, religious affiliations may have had an impact on participants’ willingness to confront their preconceived ideas.

In the Nigerian study, recognition of the capabilities of people with ID increased over time following the intervention. In the Kenyan study while there was a positive change observed in *Knowledge of Capacity* post-intervention, this change was not maintained at follow-up. An explanation for this difference might be due to the capabilities of people with ID being showcased differently in both videos, despite attempts to make the film contents the same in both countries. The Kenyan film implicitly showed the capabilities of people with ID by featuring them engaged in a range of activities. In contrast, the Nigerian film presented this information explicitly by having the individuals with ID featured state what they could do in addition to demonstrating it. This approach in the Nigerian study of combining education and indirect contact/firsthand observation of members of the stigmatised group to challenge common stereotypes of people with ID as incapable, seemed to have had a greater positive impact on participants’ attitudes than relying on indirect contact/observation alone. Furthermore, unlike *Knowledge of Capacity*, all other cognitive constructs (i.e. *Knowledge of Rights, Knowledge of Causes and Superstitious Beliefs*) were addressed by experts in both studies while images of people with ID were shown. Taking a more educational approach in this section of the films, with little input from people with ID sharing their views, appears to have resulted in the opposite effect when compared to *Knowledge of Capacity*, as little to no change was observed for these constructs, with the exception of *Supernatural Beliefs*.

These findings are in line with other studies that have shown that people with disabilities advocating for attitude change can help to promote parity over pity and be more impactful in changing attitudes in disability contexts than non-disabled others leading the charge (13). It also suggests that when trying to change beliefs within an African context, mere exposure (e.g. through images) of people with ID may not be sufficient and a level of interaction is required in order to produce positive contact outcomes. Thus, an integrated approach that positions people with ID as agents of change in public initiatives within African regions warrants further research.

Looking specifically at *Knowledge of Rights*, the difference in effects observed between both studies and at different time points may be explained with reference to Allport’s contact hypothesis. One of the images shown in the Nigerian study implicitly suggested institutional endorsement of the rights of people with ID. The image showed people with ID who were also featured in the film holding their voter’s card for the presidential election that had recently taken place in the country. This could not be replicated in the Kenyan study. Indeed, institutional support, one of Allport’s conditions, is believed to be important in producing positive contact outcomes (12). However, the magnitude of its effects has been reported to diminish when isolated from other facilitating contact conditions, conditions that were not fully met in the educational section of the intervention (12). This may explain why even though participants’ endorsement of the rights of people with ID increased, this effect was not lasting.

Irrespective of the cognitive components of attitudes and the differences identified between both films, the behavioural factors saw positive shifts that were maintained over time. In light of these findings, one might argue that while Allport’s conditions may not be necessary to reduce behavioural intent, their ability to enhance the likelihood of positive change is of importance when targeting cognitive constructs. This is because cognitive aspects of stigma within this study seemed to be more sensitive to varied contact conditions. This warrants further research.

However, the extent of noted effects in the present study comes into question when considering how attitude change based on self-report translates into actual behaviour. A meta-analysis of the intention-behaviour relationship concluded that a medium to large change in behavioural intention (*d* = 0·66) leads to a small to medium change in actual behaviour (*d* = 0·36) (27). As noted, a medium to large effect size (*d* = -0·624) was observed in Nigerian participants’ increased understanding of the capabilities of people with ID. Medium effect sizes were also observed for Kenyan participants’ increased willingness to interact with people with ID at follow-up. This suggests that brief film-based interventions have the potential to make a positive contribution to efforts to reduce hostile attitudes and treatment of people with ID in countries such as Nigeria and Kenya.

Given that the effect sizes for different subscales varied between the Nigerian and Kenyan samples, most likely reflecting the subtle differences between the two experimental films, future research should assess how to maximise the potential for change through similar interventions.

Some of the attitudinal changes observed in this study may be due to measurement effects, which can create small but transient positive shifts in attitudes that could be erroneously attributed to the intervention (28, 29). This may explain why the control groups experienced some gradual increases in positive attitudes post-intervention. However, these positive changes were not significant over time. The experimental group showed more lasting and larger attitude changes in a desirable direction than the control group.

While delivery of such interventions via the internet risks only reaching internet users, through liaison with local community groups and stakeholders, film-based interventions can readily be disseminated through events at local community level, and in schools, churches or village centres. This method of dissemination is in keeping with reports from African experts in the ID field who state that small scale face-to-face campaigns in group settings across different towns and villages in Nigeria and Kenya have a wider reach and have proven vital in tackling ID stigma (4). However, mostly education-based approaches have been used. A film-based intervention similar to the one produced in this study, which combines both education and indirect contact can be used in such efforts to: (1) increase the effectiveness of anti-stigma initiatives used; (2) standardise the interventions delivered; and (3) reduce the amount of manpower needed to carry out anti-stigma initiatives, thus opening up opportunities for low-cost, accessible approaches. This study also reinforces the usefulness of global partnerships, highlighting the different ways academics and practitioners can work together to improve the well-being of people with ID around the world. Future research should assess how to ensure impact of such interventions to enable greater reach.

### 4.1 Limitations

Despite evidence of poor internet connectivity, this barrier did not significantly affect the delivery of the intervention or data collection, as the study had a wide reach. However, the diversity of the sample was, as expected, affected by the chosen delivery method. Both samples were unrepresentative and the findings should not be generalised to the general population. In accordance with other longitudinal studies, retention rates at follow up were low in both studies, further threatening the generalisability of the findings. However, the impact of the attrition rate on the studies’ internal validity was controlled through statistical analysis. It should also be noted that the participants’ characteristics (typically educated, young, and reporting prior contact with people with ID), which were influenced by the chosen method of delivery, have all been associated with more positive attitudes in previous studies (3), and as such may have contributed to the success of the present intervention.

While statistical analysis may allow for the intention-behaviour relationship to be estimated, this relationship is limited to medium to large intervention effects (27). Effects of this magnitude were only observed for two of the six subscales across both studies. As such, the likely effects of self-reported attitude change on actual behaviour within this study remain unclear. Furthermore, measuring attitudes through self-report always poses the risk of socially desirable responding. The following steps were taken to try to control for this bias: informing participants of their anonymity, blinding them to the purpose of the study, and using a scale that includes a neutral point thus reducing a forced response. Nonetheless this is a limitation.

Using an online platform for data collection did pose the risk of encountering trolls, bots and multiple responses. While some measures were put in place to try to control for this, such as installing timestamps and having open-ended questions at the end of the survey, more could have been done to prevent for this. Future research should explore the use of more stringent procedures to uphold the quality of the responses received in online research. This may include using a completely automated public Turing test to tell computers and humans apart (CAPTCHA) and adding some quality check questions.

## 5 Conclusions

This study found that a brief, film-based e-intervention was successful in reducing stigmatising attitudes towards people with ID among Internet users in both Nigeria and Kenya. E-interventions like the integrative approach used in this study present a viable contribution to stigma reduction efforts by promoting awareness in in a manner that is cost-effective, sustainable and can reach mass audiences, while still maintaining the quality of the evaluation methods used. How to maximise the potential for attitudinal change and stigma reduction, and whether similar brief interventions can have positive effects when delivered through mediums other than the internet and thus accessible to more representative audiences are questions for future research. Furthermore, given the risk that short-term interventions may only have short lived effects, future research should assess intervention effects over a longer term.

## Data availability statement

The raw data supporting the conclusions of this article will be made available by the authors, without undue reservation.

## Ethics statement

The studies involving humans were approved by University College London Research Ethics Committee. The studies were conducted in accordance with the local legislation and institutional requirements. The participants provided their written informed consent to participate in this study.

## Author contributions

DO: Conceptualization, Formal analysis, Investigation, Methodology, Project administration, Writing – original draft, Writing – review & editing. WC: Conceptualization, Formal analysis, Investigation, Methodology, Project administration, Writing – original draft, Writing – review & editing. KS: Conceptualization, Formal analysis, Methodology, Supervision, Writing – review & editing.
