# Supplementary material for: The effect of an e-intervention on intellectual disability stigma among Nigerian and Kenyan internet users: a comparative randomised controlled trial
Source: Front Psychiatry. 2024 Mar 5;15:1331107. doi: 10.3389/fpsyt.2024.1331107 (PMC10948596; doi:10.3389/fpsyt.2024.1331107)
Supplement: Supplementary file 1 [file Presentation_1.pdf]

Supplementary Material

1. **The Attitudes toward Intellectual Disability (ATTID) Questionnaire adjusted vignettes used:**

   (Moderate ID) [African Name] is an adult with an intellectual disability. He lives at home with his parents. He dresses himself and is able to go out without getting lost but cannot take public transportation on his own. He will go on errands to the nearby shop but will not know if he has been given the right change. Jide helps with household chores such as tidying up his room, setting the table and sweeping the floor. He is able to carry on a conversation but has difficulties discussing things that are abstract or complex. Jide knows how to use the telephone and can write.

   (Severe ID) [African Name] is an adult with ID. He communicates using sounds and gestures. He is able to show by gestures that he needs to go to the toilet. Since Uche has major coordination problems, he requires constant assistance when he moves around and always has to be accompanied on outings. He also has trouble with various movements. He is able to feed himself with an adapted spoon, but he drops food.

2. **Intellectual Disabilities Literacy Scale (IDLS)**

   The three items from the fate/superstitious causes subscale that were added to the ATTID:

   In your opinion, intellectual disability might be caused by:

   1. a test from God/Allah
   2. possession by spirits
   3. punishment for past wrongdoings

Totally agree	Agree	Neither agree nor disagree	Disagree	Totally disagree	Not applicable or don't know
1	2	3	4	5	9
